# Haptic-Based Manipulation Scheme of Magnetic Nanoparticles in a Multi-Branch Blood Vessel for Targeted Drug Delivery

**DOI:** 10.3390/mi9010014

**Published:** 2018-01-01

**Authors:** Vahid Hamdipoor, Muhammad Raheel Afzal, Tuan-Anh Le, Jungwon Yoon

**Affiliations:** 1 School of Mechanical and Aerospace Engineering, Gyeongsang National University, Jinju 52828, Korea; vahid.hamdipoor@gmail.com (V.H.); raheel379@gmail.com (M.R.A.); 2 School of Integrated Technology, Gwangju Institute of Science and Technology, 123 Cheomdan-gwagiro, Buk-gu, Gwangju 61005, Korea; tuananhtbbk@gmail.com

**Keywords:** magnetic nanoparticles, multi-branch vessels, haptics, guidance, electromagnetic actuator, targeted drug delivery

## Abstract

Magnetic drug targeting is a promising technique that can deliver drugs to the diseased region, while keeping the drug away from healthy parts of body. Introducing a human in the control loop of a targeted drug delivery system and using inherent bilateralism of a haptic device at the same time can considerably improve the performance of targeted drug delivery systems. In this paper, we suggest a novel intelligent haptic guidance scheme for steering a number of magnetic nanoparticles (MNPs) using forbidden region virtual fixtures and a haptic rendering scheme with multi particles. Forbidden region virtual fixtures are a general class of guidance modes implemented in software, which help a human-machine collaborative system accomplish a specific task by constraining a movement into limited regions. To examine the effectiveness of our proposed scheme, we implemented a magnetic guided drug delivery system in a virtual environment using a physics-based model of targeted drug delivery including a multi-branch blood vessel and realistic blood dynamics. We performed user studies with different guidance modes: unguided, semi virtual fixture and full virtual fixture modes. We found out that the efficiency of targeting was significantly improved using the forbidden region virtual fixture and the proposed haptic rendering of MNPs. We can expect that using intelligent haptic feedback in real targeted drug delivery systems can improve the targeting efficiency of MNPs in multi-branch vessels.

## 1. Introduction

Magnetic drug targeting has gained noteworthy interest among scientists and researchers during the last two decades due to a potential increase in the amount of medicine intake in order to substantially improve therapy efficiency. Magnetic drug targeting is a technique that utilizes magnetic nanoparticles (MNPs) to carry drugs within a blood stream and guides them using an external magnetic force. Target position in the Magnetic drug targeting can be a tumor or an infectious region or any malignant tissue [1,2]. Magnetic drug targeting efficiently targets the malfunctioned region, while traditional therapeutic methods affect healthy organs as well. Consequently, it is more effective and has fewer side effects [3,4]. However, many challenges for successful implementation of a targeted drug delivery system remain unsolved. The primary challenge in targeted drug delivery is precisely steering MNPs to the target region and especially to the deep tissues. Existing permanent magnets cannot capture the MNPs into deep regions inside the body [5]. In addition, real time tracking and feedback control systems are essential to steer nano-particles precisely [5]. For real time tracking, magnetic resonance imaging (MRI)-based systems to steer microparticles in the blood vessel have been introduced by [6,7]. In [8], ultrasound feedback is used to track microparticles inside blood vessel. To achieve a real-time steering scheme, we developed a Magnetic Particle Imaging system (MPI) for real time tracking and steering of specific MNPs [9,10,11]. Position information of MNPs received from the MPI monitoring system can be used to allow a real-time closed-loop motion control in targeted drug delivery.

For the feedback control in targeted drug delivery, most of the studies focus on a micro size single particle control in vitro and in vivo [12,13,14]. However, precise steering of several nanoparticles has more difficulties than that of a single particle due to their dispersions during manipulation. Optimal feedback control of the distributed nanoparticles is discussed in [15,16]. The location of most blood vessels, the blood flow velocities of vessels, and many other biological factors are not identified in general or for each person [15]. Beyond many issues caused by the complexities and variations of the human body, the performance of the autonomous control in a targeted drug delivery systems is also restricted by the natural tendency of the magnetic fields to disperse magnetic particles moving under applied forces. This actuation issue gradually decreases the concentration of particles when a group of magnetic nanoparticles moves inside a magnetic field and consequently makes the effective control of particles very difficult [16]. Due to these modeling and actuation complications of MNPs, automatic approaches for steering MNPs may not be feasible solutions in drug targeting applications. Thus, providing a human operator in the control loop would be robust in terms of modeling uncertainties and actuation issues during tele-manipulation of MNPs, which would increase safety, responsibility and public acceptance of a system for drug delivery applications of a human body [17]. In such a case, a tele-operated system using a haptic device can offer an intelligent and intuitive interaction with the remote environment during manipulation.

Unlike contact-based micro and nano manipulations such as Atomic Force Microscopy, there are few studies with haptic feedback in non-contact micro and nano manipulations. Existing non-contact systems with haptic feedback are mainly on magnetic tweezers and optical tweezers [18,19,20,21]. Haptic force feedback is not only beneficial to render the mechanical properties of a remote environment, but it can also be useful by providing navigation information to the human operator [22]. Providing navigation information in teleoperation to assist an operator can be implemented by virtual fixtures known as active constrains [23]. The term “virtual fixture” refers to a general class of guidance modes implemented in software, which helps a human-machine collaborative system accomplish a specific task by constraining a movement into limited regions and/or affecting a movement along desired trajectories [24]. There are two types of virtual fixtures: guidance virtual fixtures and forbidden region virtual fixtures. Forbidden region virtual fixtures constrain movement into a certain regions [25]. In order to avoid some region inside the blood vessel in steering of MNPs, the concept of forbidden region virtual fixtures seems to be well-suited to the targeted drug delivery applications for combining a haptic feedback scheme. Even though extensive researches have been carried out on the application of virtual fixtures in the micro manipulation [26,27,28], all of these studies are considering single micro particle or single molecule. However, the interaction approach for steering of multi nanoparticles in targeted drug delivery has not been discussed yet and it is probably different from the manipulation of single object.

To the best of the authors’ knowledge, there are no existing researches on using virtual fixtures and multi-particle haptic rendering for nanoparticles manipulation. The main contributions of this paper are adding a human operator to the control system of a targeted drug delivery system using a haptic device and proposing a new scheme for haptic rendering of multi particles to calculate force feedback to the user and utilizing a virtual fixture for more efficient steering of MNPs inside a blood vessel. To examine the efficiency of the proposed scheme, we developed a virtual environment using a physics-based model of a targeted drug delivery system, performed user studies with the different assistance modes and investigated the effects of haptic feedback and virtual fixture for guidance of MNPs.

## 2. Materials and Methods

### 2.1. Magnetic Nano-Manipulation for Targeted Drug Delivery

**Figure 1:**
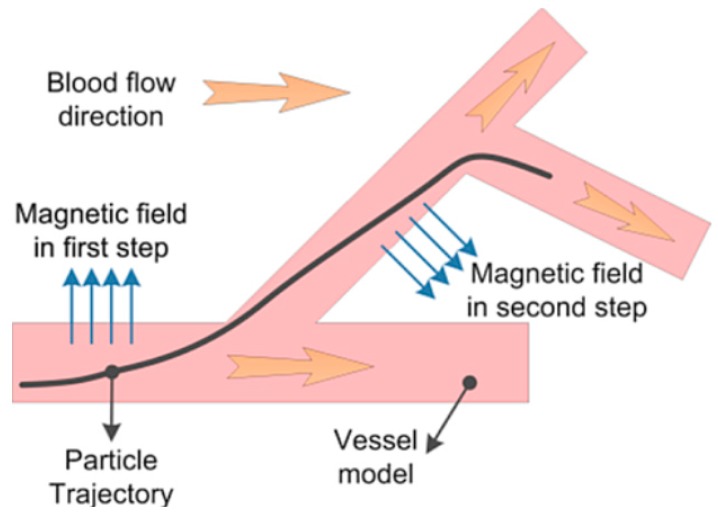
Guidance scheme of magnetic nanoparticles at blood vessel by applying magnetic force.

When injected in to the blood vessel, the MNPs are assumed to be carried along by the blood flow. We use electromagnets to steer these MNPs, by altering their radial position as they move with the blood. MNPs are directed towards the desired exit at an upcoming bifurcation by steering them to the appropriate region of the vessel before they arrive at the bifurcation. A representation of this guidance scheme is shown in Figure 1.

There are several forces which are acting on nanoparticles inside blood vessels such as hydrodynamic drag force, inertia, buoyancy, gravitational and particle-particle interaction. However, only dominant forces are considered in the proposed tele-nano-manipulation system for simplicity of computation and real-time interaction with a haptic device. Using Newton’s law, the trajectory of a particle in fluid by total force ***F*** can be described as:

(1) $$m_{p}\frac{dV_{p}}{\mathrm{dt}}=F$$

where, $m_{p}$ and $V_{p}$ are the mass and velocity of the particle, respectively. Among the many forces acting on moving nanoparticles in blood vessels, hydrodynamic drag force and magnetophoretic force are major contributors. The other forces can be ignored in a blood vessel model because they are several orders of magnitude weaker than the magnetic force. Ignoring those low impact forces, the total force on a particle can be expressed as follows:

(2) $$F=F_{\mathrm{MAP}}+F_{\mathrm{drag}}$$

The hydrodynamic drag force ($F_{\mathrm{drag}}$) is given by Stoke’s law as

(3) $$F_{\mathrm{drag}}=-6\pi \eta R\left(V_{p}-V_{f}\right)$$

where, η is the fluid viscosity, R is the particle radius, $V_{p}$ is the particle velocity and $V_{f}$ is the fluid velocity. Also the magnetic force $F_{\mathrm{MAP}}$ can be given as:

(4) $$F_{\mathrm{MAP}}=\frac{4}{3}\pi \mu_{0}R^{3}M\left(H\right).\nabla H$$

where, $\mu_{0}$ is permeability of free space, **M** is net magnetic polarization, **H** is a magnetic intensity, and ∇H is the gradient of the magnetic intensity. Based on Equations (1)–(4), we can get particle trajectory equation as follows;

(5) $$m_{p}\frac{dV_{p}}{\mathrm{dt}}=\frac{4}{3}\pi \mu_{0}R^{3}M\left(H\right).\nabla H-6\pi \eta R\left(V_{p}-V_{f}\right)$$

If the particle is magnetically soft and the magnetic field is strong, then the particle will saturate so that **M**(**H**)→M_sat_. By solving this equation, the trajectory of the particles could be calculated.

Since the fluid velocity profile in a channel is curved, it is the highest at the centerline and is zero at the walls. For Newtonian fluids in straight channels at steady state, this curved profile is parabolic. Blood, however, is a non-Newtonian fluid due to the presence of the clotting protein fibrinogen which causes red blood cells to aggregate at low shear rates. Such a profile can be fit empirically by:

(6) $$V=V_{Bmax}(1-\left(\frac{r}{R}{)}^{\xi }\right)$$

where *V* is the blood velocity (m/s) in the axial direction , $V_{Bmax}$ is the maximum centerline blood velocity (m/s), *r* is the radial location (m), R is the radius of the vessel (m), and ξ is a constant for a particular profile. We chose a value of ξ = 9 for our targeted drug delivery system. This equation obviates the need to solve the Navier–Stokes equations for the flow profile. In rat vessels, the minimum centerline blood velocity is in the order of 0.1 mm/s, in humans it is around 0.5 mm/s [4].

### 2.2. Virtual Tele-Nano-Manipulation System

#### 2.2.1. System Overview

**Figure 2:**
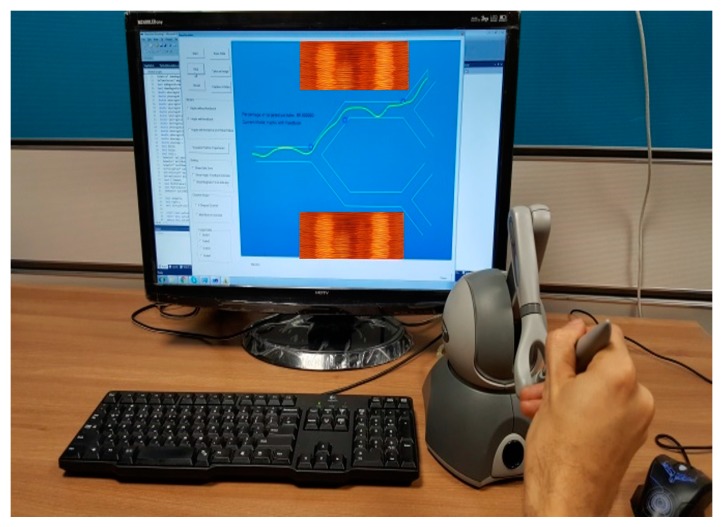
Virtual tele-nano-manipulation system.

Due to advances of virtual reality (VR) technologies, virtual environment simulations in a manner similar to the physical tele-nano-manipulation system can replicate real in vivo experiments without implementing whole hardware systems, and we can get an objective analysis of haptic guidance effects during the tele-operated interactions of a user for manipulations of MNPs inside virtual environments. Our proposed system consists of a virtual environment, a haptic device and a user as shown in Figure 2. The developed virtual environment is a 2D simulation platform, which includes a graphic engine, a simulation engine and a haptic rendering engine.

**Figure 3:**
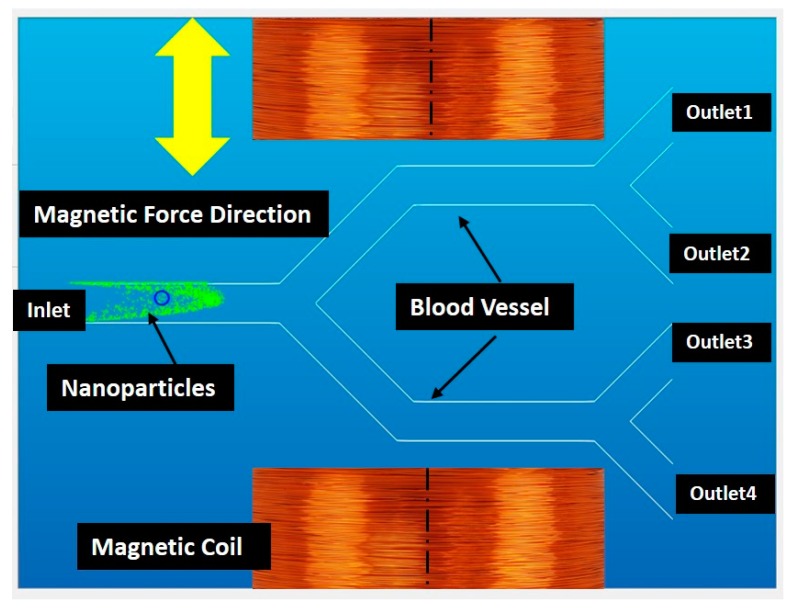
Elements of the virtual environment.

The virtual environment is developed with C++ programming language and Open Inventor API (Thermo Fisher Scientific, Waltham, MA, USA). Figure 3 shows a screen shot of our virtual environment. It includes a user interface and a graphical view of a multi-branch blood vessel. In the graphical view, a 2D model of blood vessel has been modeled and shown to the user. The blood vessel has one inlet (left side) and four outlets (right side). MNPs are injected in the inlet; they move towards the outlets by a defined blood flow with a drag force and can change their positions with a magnetic guiding force. There are two magnetic coils to indicate the position and direction of the magnetic force.

PHANTOM Omni haptic device (SensAble Technologies, Inc., San Francisco, CA, USA) is used to interact with the virtual environment. It is a three degrees of freedom device that provides three input coordinates in a form of position and three outputs as force feedback coordinates. The characteristics of the Omni device are as follows; maximum force of 3.3 N, translational workspace of 380 mm × 267 mm × 191 mm and rotational workspace of 297°, 260°, 335° (yaw, roll, pitch). OpenHaptics API (3D Systems, Rock Hill, SC, USA) is used to provide reliable communication between the virtual reality software and the haptic device. OpenHaptics (3D Systems, Rock Hill, SC, USA) provides the most basic functions for developing haptic applications. The haptic rendering loop runs at 1 kHz.

#### 2.2.2. System Architecture

**Figure 4:**
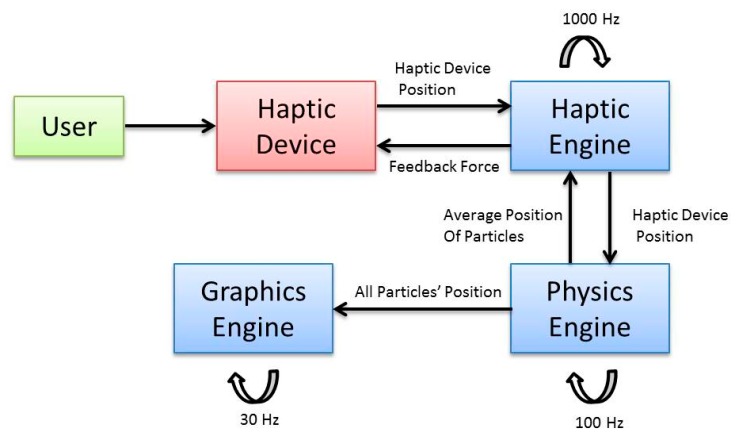
System architecture and data flow of different modules.

There are two kinds of processes inside the system, real time processes and offline processes. The offline processes deal with the receiving of experiment parameters and calculating forbidden region virtual fixtures for the manipulation of MNPs based on a selected target outlet of a virtual blood vessel. The real time processes comprise of a physics engine, a graphic engine and a haptic engine. An overview of system architecture and data flow and update rate of each engine is presented in Figure 4. Graphics engine receives particles’ position data and updates their position at 30 Hz. Acquisition time of the MPI system, which is the proposed tracking method in this paper, can reach 21 ms (50 Hz) [29]. The physics engine sends particles’ position to the haptic engine and graphics engine at the rate of 100 Hz. The haptic engine receives average position of particles from physics engine and sends haptic device position to the physics engine and force feedback to the user through haptic device at the rate of 1000 Hz.

During virtual tele-nano-manipulation operations, injected nanoparticles are moving by a blood stream and the operator should position them near a desired outlet using the haptic device. To position nanoparticles near the desired outlet, the user changes the magnitude and direction of magnetic force acting on MNPs. User is responsible for keeping particles in the right track and preventing particles from dispersing inside the blood flow. Also, the user should pay attention to the force feedbacks exerted from the haptic device to change the magnetic force during the manipulation.

### 2.3. Haptic Interaction for Manipulations of MNPs

#### 2.3.1. Overview

**Figure 5:**
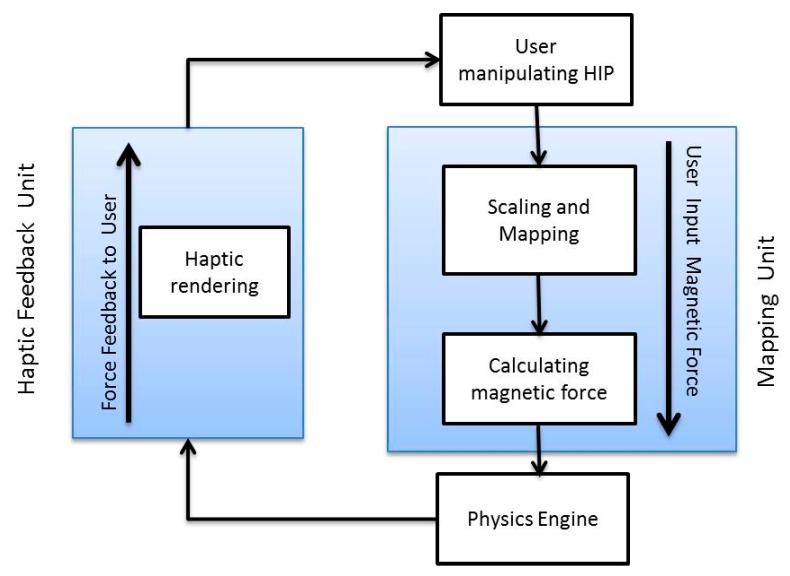
Haptic engine unit for tele-manipulation of magnetic nanoparticles (MNPs).

The presented research intends to boost the human-in-the-loop nano-manipulation for targeted drug delivery through haptic interaction. Since the haptic device is inherently a bilateral device, it is essential that two separate units should be considered for effective haptic interaction. The first unit is the mapping framework which enables the user to steer MNPs by changing magnetic force. The second unit is the haptic rendering which exerts a haptic force to the user in the case of contact of MNPs to the virtual walls. The physics engine acts as an interface between the two units of the haptic engine. The mapped magnetic force from the haptic device movement acts as an input to the physics engine and MNPs average position acts as an input for the haptic rendering unit. One of the challenging problems to integrate the force feedback is how the physics engine satisfies the high update frequency (1000 Hz) of haptic rendering for stable force feedback. Since the frequency of the physics simulation is limited to 100 Hz in the physics engine, the physics engine and haptic engine are made to run in an asynchronous execution [30]. Figure 5 illustrates the two main haptic units of a tele-nano-manipulation system.

#### 2.3.2. Physics Engine for Particles Simulations inside Blood Vessels

**Figure 6:**
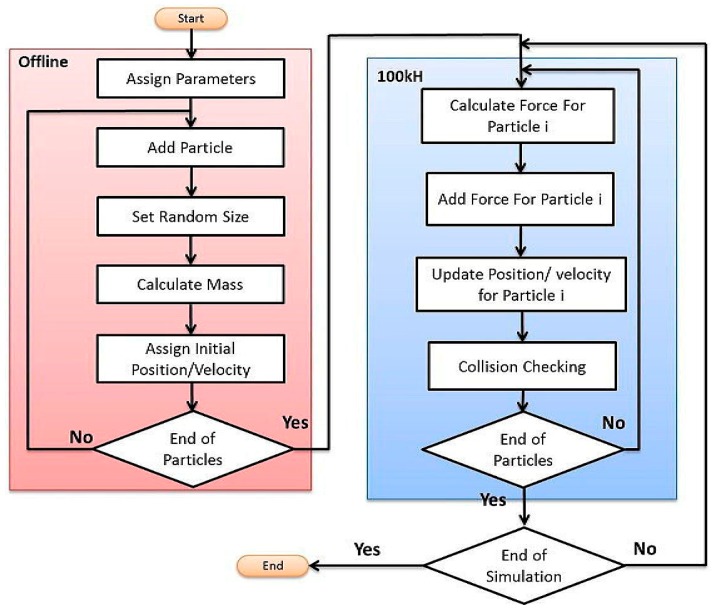
The flow chart of simulation steps at physics engine.

A physics engine is responsible for generating the real-time position of nanoparticles at each time step. C++ language is used to make our physics engine. Particle based modelling is the basis of physics engine simulations. Particle based modeling uses a particle system which typically includes massive collection of particles generated from one or more locations in space. The motion of particles is determined by external forces acting on each particle [31]. Various steps of simulation at the physics engine are shown in Figure 6. After initializing the particle system, forces acting on each particle should be calculated at each time step using Equation (1), and then the positions of particles can be achieved through numerical integration. If there is no collision between a particle and a blood vessel, the same position is kept; otherwise, the position of the particle is recalculated after collision check with the channel walls. The physics engine is running at 100 Hz update rate. Table 1 presents the parameters used in the simulation studies. The diameters of MNPs are chosen randomly between 400 nm and 1000 nm. Even though this variation in size of the nanoparticles changes the amount of magnetic force received by each particle, it makes the simulation more realistic.

#### 2.3.3. Mapping Framework

In this section, a position-force mapping framework in presented. This framework enables a user to control nano-manipulator intuitively through a haptic device. Since the position of MNPs is determined by a blood flow and a magnetic force, the user cannot have full control over the position of MNPs. Moreover, since the relationship between the magnetic force and the displacement of nanoparticles is not linear and since it depends on many parameters, mapping from the linear movement of haptic interaction point (HIP) to the nonlinear displacement of MNPs is almost impossible. In this research, we suggest a novel position to force mapping approach which maps the HIP movement in a vertical direction of the haptic device to the magnetic force for more realistic nano-manipulation.

**Figure 7:**
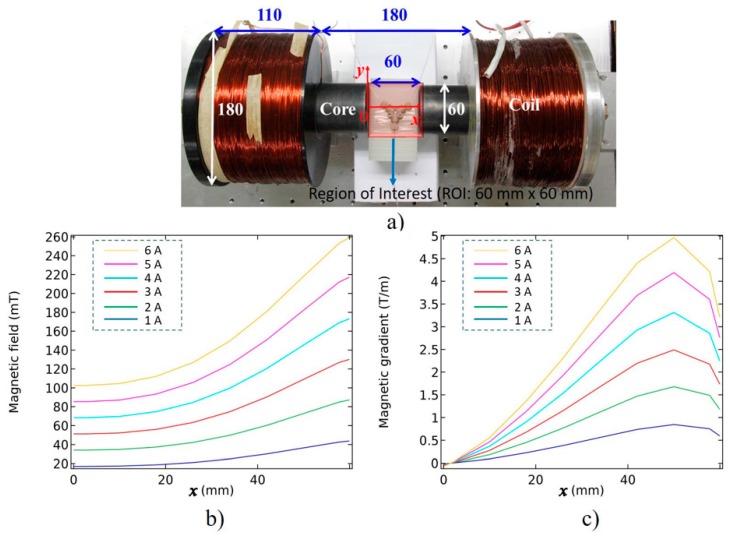
Electromagnetic actuation system applied to the developed virtual reality (VR) simulator. (**a**) Electromagnetic nano actuation system setup and its different parameters (all the dimensions are in mm). (**b**,**c**) Spatial distribution of the magnetic field and magnetic gradient in practice, respectively; when current from 1 A to 6 A is applied to the right coil. We used the Bell-5180 Gaussmeter (Transcat, Inc., Rochester, NY, USA) to record this data.

**Figure 8:**
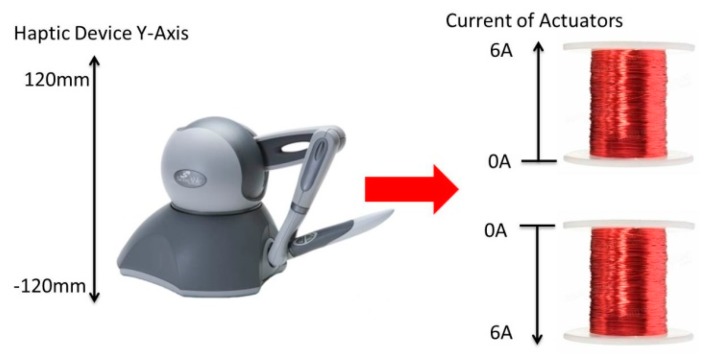
Mapping from workspace of the haptic device to force of the electromagnetic actuator.

**Figure 9:**
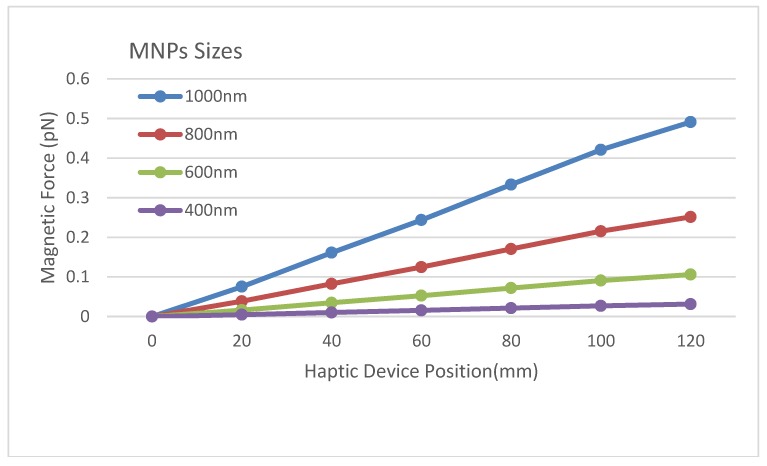
Resultant magnetic force on a particle from haptic device movement.

The main goal of our tele-nano robotic system is to guide MNPs inside the virtual blood vessel by applying appropriate magnitudes and directions of magnetic force to change the positions of nanoparticles inside the vessel. In our system, we mapped the deviation amount of Y-axis position from a neutral home position of the haptic device to the input current of electromagnetic actuators. The parameters and formulae of our developed virtual system are based on our real electromagnetic actuation system (Figure 7) for magnetic nanoparticles, whose targeting efficiency had been studied through simulations and experiments [32,33,34,35]. The relationship between the input current and magnetic field intensity, and magnetic field gradient at the Region of Interest (ROI) is presented in Table 2 (obtained from the experimental data of our previous study [35]). By using Equation (4), we can compute the amount of magnetic force on each particle given the magnetic field gradient. Because our real actuators have a magnetic force in one axis, we need to map one dimension of the haptic device to a current value and then apply the magnetic field gradient associated with it from Table 2. Besides, results in [12] show that a minimum magnetic gradient of 0.7 T/m would be enough for the purpose of targeted drug delivery. Thus, our system which has a maximum gradient of 2.8 T/m at ROI center can navigate the magnetic nanoparticles. In this paper, we have utilized the same magnetic gradient as we had used for in vivo experiments on mice [36]. Results in [36] show that MNPs successfully reached the mouse’s brain and the evidence of blood-brain barrier crossing was also observed. Figure 8 illustrates the mapping between the workspace of the Phantom Omni mapping from −120 mm to +120 mm to the current value 0 A to 6 A for each actuator. The negative sign will prompt the direction change of the magnetic force by changing the actuator current. In this simulator, the sizes of nanoparticles are chosen randomly between 400 to 1000 nm. The resultant magnetic force, generated from the movement of haptic device, on a particle is shown in Figure 9. As seen in Figure 9, the magnetic forces on a particle are quite weak. However, the MNPs always exhibit aggregation phenomenon inside a magnetic field. For a realistic simulation, a mean aggregate size was considered based on [37]. As explained in [37], the particles grow rapidly into a “local aggregate” at this length scale (<1000 nm). The diameter of a “local aggregate” particle can increase to about 10–100 times when compared with the diameter of a single particle. Thus, a force factor corresponding to a particle with a diameter increased by a factor of 10 has been added to make the simulation more realistic.

#### 2.3.4. Forbidden Region Virtual Fixtures inside Blood Vessels

Previous researches show that using a virtual fixture can significantly increase the guidance performance of an operator. In this paper, Forbidden Region Virtual Fixtures are introduced to assist a user during manipulation of MNPs. Since both the user’s position input and haptic feedback occur at the HIP due to the inherent bilateral nature of the haptic device, and the maximum force for the feedback of Phantom Omni is less 3.3 N. The user can override haptic forces to perform independent control decisions. In such a way, the user can decide whether continue the manipulation through the virtual fixture suggestions and ultimately decide to choose an appropriate control action during the manipulation [28].

**Figure 10:**
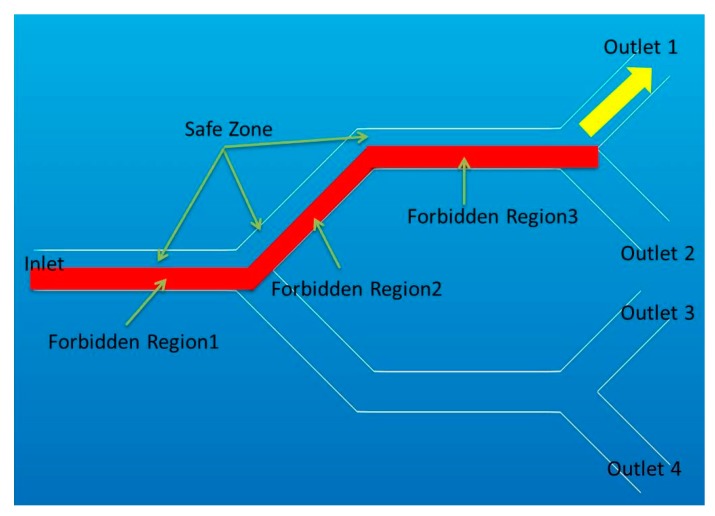
Forbidden region and safe zone for a multi-channel blood vessel with outlet 1 as target outlet.

To define the forbidden region virtual fixtures for targeted drug delivery, we use the concept of a “safe zone”. In [32], we introduced the concept of a safe zone for targeted drug delivery inside blood vessels. The safe zone is a part of the vessel that all particles located inside that region will reach a correct outlet, even when there are no external forces applied to the particles. Therefore, we need to steer all the particles into the safe zone. The forbidden region is the exactly opposite side of the safe zone. It is a region that particles should avoid entering for correct guidance. We consider a virtual wall which is located between the forbidden region and the safe zone as a vertical plane in the middle of the blood vessel. Figure 10 shows the forbidden regions for a multi-branch targeted drug delivery system.

#### 2.3.5. Haptic Rendering for Multi Particles in a Multi-Branch Blood Vessel

**Figure 11:**
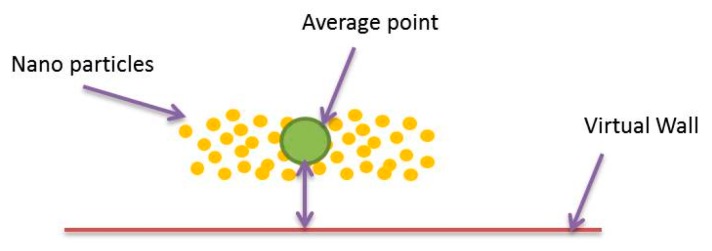
Averaging nanoparticles to render force feedback for multi particle drug delivery system.

The aim of Forbidden Region Virtual Fixtures is to prevent nanoparticles from entering into undesired areas or alarming a user about entering those areas. We consider a virtual wall at each branch, which acts as a unilateral spring. For stability considerations, we have limited the stiffness of the virtual wall in a stable range. There are two major challenges in haptic guidance for nano-manipulation with multi particles in a multi-branch for targeted drug delivery systems, firstly, controlling simultaneously multiple nanoparticles and secondly, dealing with a multi-branch blood vessel. Our approach for the first challenge is using an average position of nanoparticles in the vessel and preventing it from entering into the forbidden region (see Figure 11).

**Figure 12:**
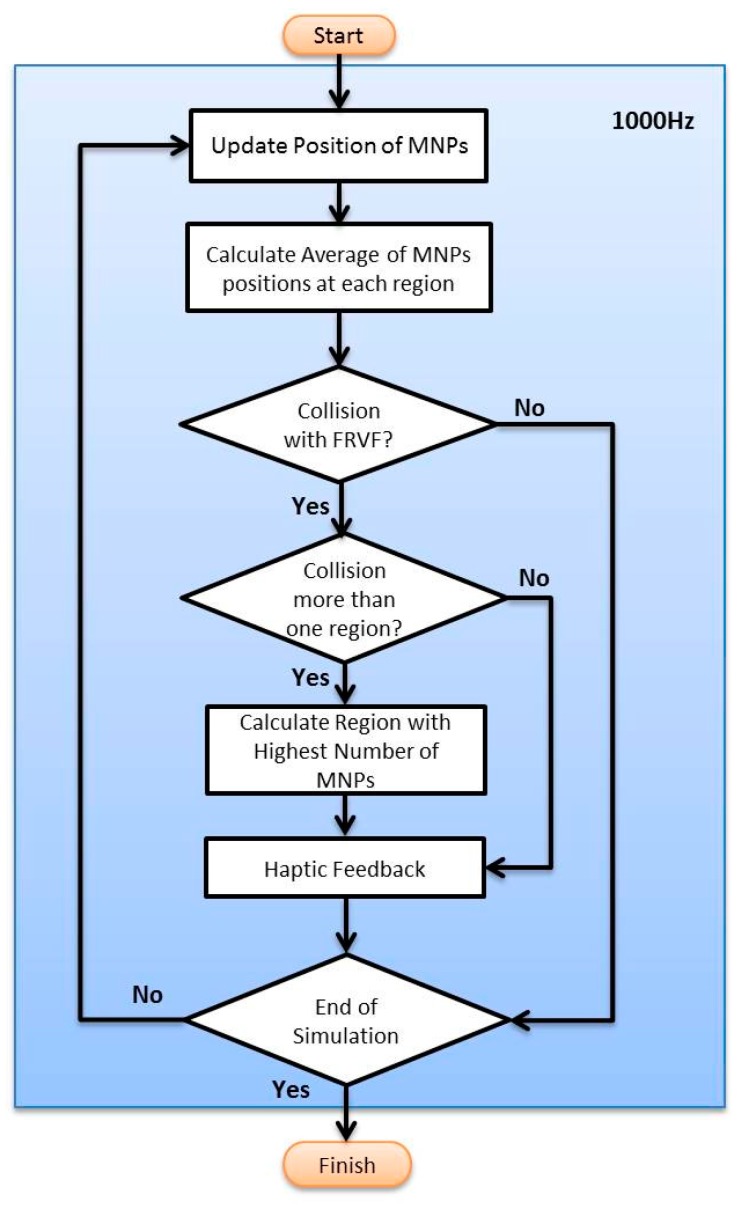
Flow chart of main algorithm for haptic rendering.

The second challenge happens because the vessel is composed of a multi branch. As mentioned earlier, MNPs tend to disperse under magnetic force. This issue makes the first solution ineffective. To overcome this challenge, we propose a regional averaging for haptic rendering with multi-particles. In the regional averaging, we only calculate the average of MNPs’ positions at a separate region for each branch. If the regional average position of MNPs passes the virtual wall, a feedback force is rendered to a user through the haptic device (see Figure 10). In the cases where two different regional averages pass the virtual wall but their directions are opposite to each other, we give a priority to the regional average that has more number of particles in the region. The amount of the feedback force applied to the user is calculated based on:

(7) $$\vec{F}=\mathrm{kD}\vec{n}$$

where $\vec{n}$ is the direction vector and because we only have actuation in Y direction, it is equal to (0,1,0) or (0,−1,0), and D is the penetration of equivalent circle to the virtual wall, and k is the stiffness of the virtual wall. The flow chart of the force feedback rendering with multi particles for nano-manipulation in a multi-branch system can be seen in Figure 12.

### 2.4. User Studies for the Virtual Tele-Nano-Manipulation

An experiment was designed to evaluate the efficacy of the virtual guided tele-nano-manipulation simulation of MNPs with haptic feedback and the virtual fixture from the suggested haptic rendering schemes in the Section 2.3. The process of steering nanoparticles from the injected position to the desired outlet of a multi-channel blood vessel was the basic task to be performed by the user participating in the experiment. Simulation schemes were performed in three modes in five different blood velocities. The modes are classified as unguided (U), semi virtual fixture (SVF) and full virtual fixture (FVF). In all modes, user should try to steer nanoparticles to the desired outlet as much as possible. After starting the tele-nano-manipulation simulation, particles are carried away by a blood flow in the meanwhile a user should steer nanoparticles by changing the magnitude of the magnetic force, and the directions and positions of particles toward the correct bifurcation. During the nano-manipulation, the user can receive certain assistance features based on the selected operating mode.

In the unguided mode, the user changes the magnetic force acting on particles without receiving any assistance. Only visual feedback from the simulator is assisting the user to steer nanoparticles to the desired outlet. In this mode, the direction and magnitude of magnetic force are fully under the control of the user. In the semi virtual fixture mode, force feedback only exists near the vessel walls. Since the blood velocity near the vessel walls is zero, nanoparticles tend to stick or move slowly in these areas. To make the user aware of this situation, whenever particles move toward the vessel wall, a force feedback in opposite direction is sent to the user and since the amount of feedback force is not high (less 1 N), the user can either override the force or comply with it to steer nanoparticles. In the FVF mode similar to SVF mode, we have force feedback near the vessel walls. Furthermore, there is another force feedback whenever particles try to leave the safe zone area. Similarly, while feeling haptic force, the user can override or accept the guidance force. 

**Figure 13:**
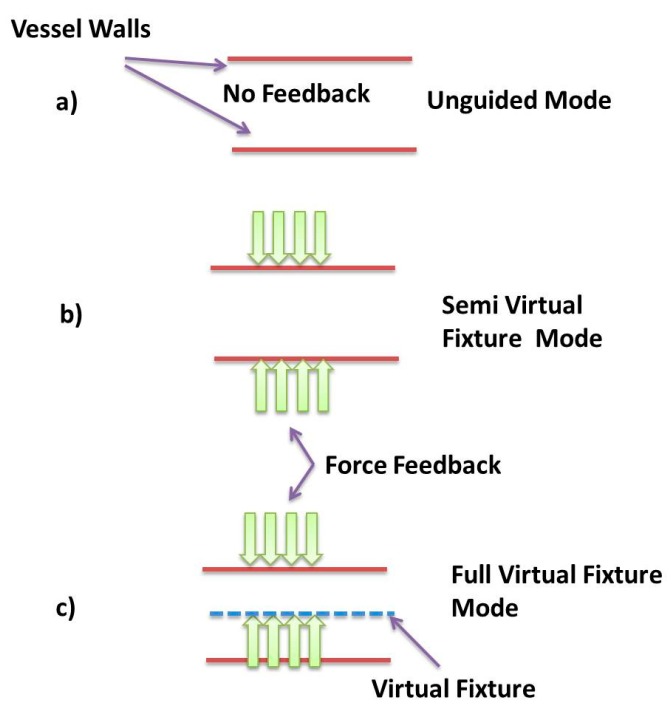
Difference between working modes of virtual reality system. (**a**) Unguided mode; (**b**) Semi virtual fixture mode; (**c**) Full virtual fixture mode.

Figure 13 illustrates these three different modes in detail. In the unguided mode, there is no haptic feedback and the haptic device is only used as a joystick to steer nanoparticles. The semi virtual fixture mode only provides the feedback at vessel walls, where the blood velocity is zero and the particles tend to stick at those locations and hence decrease the efficiency of targeting. The full virtual fixture mode provides feedback at both the safe zone boundary and the vessel wall. The average point of particles at the dominant area is shown by a blue circle in the simulator and whenever there is a haptic feedback, the color of the circle color becomes red.

Since nanoparticles are carried by a fluid and guided by a haptic device at each branch bifurcation, the goal of the experiments is to evaluate the percentage of the correctly targeted MNPs in each feedback mode of the virtual system. Twelve participants (9 men and 3 women with an average age of 28) performed manipulation of MNPs in three different modes of the virtual system at five different velocities. Participants did not have any prior experiences of working with a haptic device; therefore, each participant was allowed to try the system a few times to become familiar with the virtual environment and tele-nano-manipulation process. To eliminate or reduce the effect of learning on the results of the study, the experiment order was randomized. The desired outlet has been shown in the Figure 13 and success rate is recorded based on this outlet.

## 3. Results

Experimental data gathered in the user study was statistically analyzed to evaluate the effects of various haptic cues and blood velocities on the success rate of nanoparticles reaching the target outlet. We performed two-way Analysis of Variance (ANOVA). Factors were defined as haptic cue (3 levels: UG; SVF; FVF) and blood velocity (5 levels: 1; 2; 3; 4; 5). In addition, Mauchly’s test of Sphericity was used and Greenhouse-Geisser corrections were applied in case of its violation. Significance level (*α*) was set at *p*-value < 0.05. The output of two-way ANOVA is presented in Table 3. Post hoc tests were conducted using the Bonferroni correction method. All statistical analysis was performed using SPSS V20.0 (IBM Corp., Armonk, NY, USA).

**Figure 14:**
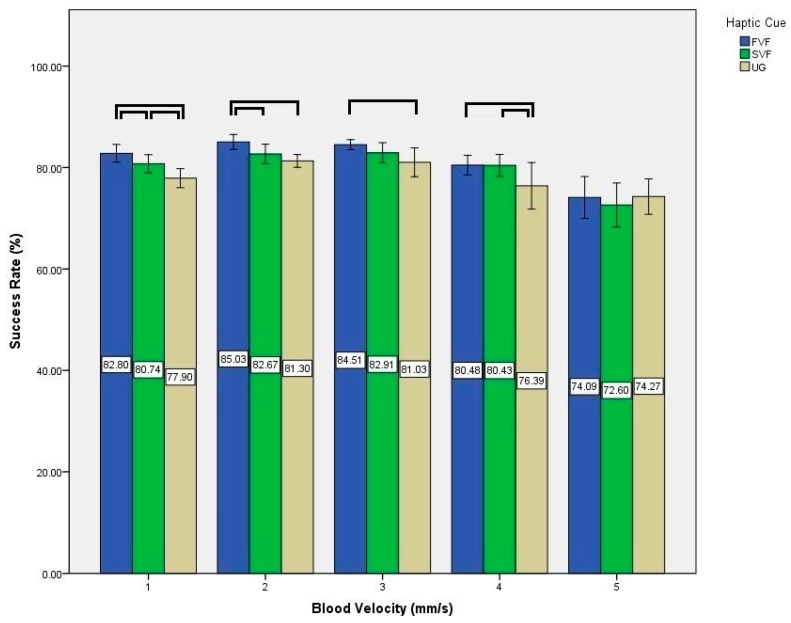
Effect of haptic cues on success rate at multiple blood velocities. Here, each ﹇ represents a statistically significant difference between the underlying two conditions.

**Figure 15:**
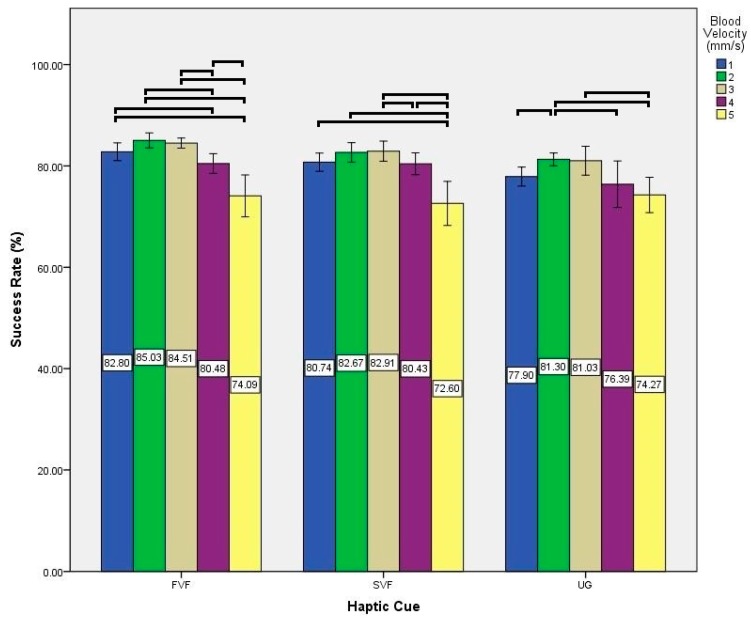
Effects of a blood velocity on success rate during various haptic cues. Here, each ﹇ represents a statistically significant difference between the underlying two conditions.

Simple main effects were tested for post-hoc analysis due to significant interaction between haptic cue and blood velocity on success rate (see Figure 14 and Figure 15).

At blood velocity of 1 mm/s, significant differences existed between UG and SVF (*p*-value = 0.005), UG and FVF (*p*-value < 0.001), and SVF and FVF (*p*-value = 0.008). At blood velocity of 2 mm/s, significant differences existed between UG and FVF (*p*-value < 0.001), and SVF and FVF (*p*-value = 0.012). At blood velocity of 3 mm/s, significant difference existed between UG and FVF (*p*-value = 0.009). At blood velocity of 4 mm/s, significant differences existed between UG and SVF (*p*-value = 0.009), and UG and FVF (*p*-value = 0.24).

While utilizing UG haptic cue, significant differences existed between various blood velocities; between 1 and 2 (*p*-value = 0.002), 2 and 4 (*p*-value = 0.002), 2 and 5 (*p*-value < 0.001), and 3 and 5 (*p*-value < 0.001). On the other hand, while utilizing SVF haptic cue, significant differences existed between various blood velocities; between 1 and 5 (*p*-value = 0.001), 2 and 5 (*p*-value < 0.001), 3 and 4 (*p*-value = 0.008), 3 and 5 (*p*-value < 0.001), and 4 and 5 (*p*-value = 0.007). Also while utilizing FVF haptic cue, significant differences existed between various blood velocities; between 1 and 4 (*p*-value = 0.024), between 1 and 5 (*p*-value < 0.001), between 2 and 4 (*p*-value < 0.001), between 2 and 5 (*p*-value < 0.001), between 3 and 4 (*p*-value = 0.001), between 3 and 5 (*p*-value < 0.001), and between 4 and 5 (*p*-value = 0.009).

## 4. Discussion

Haptic virtual fixtures provide valuable assistance to the user during micromanipulation [28]. Utilizing haptic force feedback significantly improved the performance of a micro teleoperation system for self-propelled microjet inside fluid [17]. Herein, we proposed a tele-manipulation system of MNPs leveraging forbidden region virtual fixtures to enhance the guidance performance for targeted drug delivery systems. To evaluate the performance of targeted drug delivery systems, the percentage of successfully targeted particles in a desired bifurcation have been studied recently [32,33,34]. In order to evaluate the performance of the proposed virtual tele-manipulation system of MNPs, we conducted an experiment with different guidance conditions to show efficacy of using kinesthetic force feedback and virtual fixtures in magnetic drug delivery applications. In these experiments, haptic feedback is used to give navigation information to the user to steer nanoparticles more efficiently to the desired location.

Experiments showed that using haptic feedback and virtual fixture can improve the targeting performance significantly at lower velocities (≤4 m/s). Since the lowest blood velocity in humans is 0.5 mm/s [4], using haptic feedback and virtual fixture can be used in relatively wide range of velocities between 0.5 mm/s to 4 mm/s in humans. Nonetheless, in higher velocities (>4 mm/s), there is no significant difference between applying haptic feedback and not using it. In the lower velocities (*v* = 1 mm/s, *v* = 2 mm/s), FVF mode is significantly better than SVF mode, which shows that combining the virtual fixture for the safe zone and the virtual fixture for channel wall can improve the success rate significantly. However, in the velocities *v* = 3 mm/s and *v* = 4 mm/s there is no significant difference between SVF and FVF modes while both modes perform significantly better than UG mode. This fact indicates that using the virtual fixture for a safe zone is not as effective as the virtual fixture for a channel wall. Success rate is *v* = 1 mm/s because of existence of more time to steer particles to the desired location was expected to be higher than other velocities; however, surprisingly, it is lower than *v* = 2 mm/s and 3 mm/s. This may happen because of the sticking phenomenon at the blood vessel walls where the blood velocity is too slow [32]. It is also worth noting that the provided haptic force sometimes tends to generate abrupt changes in the position of the haptic device due to abrupt changes in the particles regions or sudden collisions with the virtual fixture, which should be considered during the manipulation. To represent aggregation effects during operation in this simulator, a force factor has been added to make the simulation more realistic. However, a 3-axis actuation and aggregation should be considered in the future studies.

Our ultimate goal is to implement a human-in-loop based navigation system for targeted delivery of magnetic nanoparticles inside a body. Since tumor cells may have different physiological characteristics compared to normal cells, it is quite difficult to ensure open-loop magnetic control without monitoring magnetic nanoparticles. In this research we proposed to implement a human-operated guidance scheme in targeted drug delivery (TDD) application, similar to a surgery robot [25], to increase the targeting efficiency despite the modeling uncertainties. Therefore, the delivery performance will not be degraded with tumor cells or different disease conditions. However, the nanoparticles should be carefully designed to release the drug when the particles reach a target region with respect to different disease conditions. To apply our proposed method at different organs, the maximum blood flow rate and the shape of the vessel in that specific area should be extracted by the latest biomedical technologies [38]. Then, the required magnetic force can be calculated and generated for effective guidance. Our system has been verified for small animals [36]. However, it cannot be directly scaled up for human application. The required magnetic gradient varies according to the target depth in the body, i.e., the deeper the target, the higher the required magnetic gradient [5]. For human application, the supplied power and voltage of the electromagnetic actuation system need to be increased. Besides, the coil diameter also needs to be increased to achieve a higher gradient. Although the application of a static magnetic field is considered safe for humans [39], the magnetic gradient can pose potential health risks including peripheral nerve and cardiac stimulation to the subjects [40]. Even though the high power required can be supplied using currently available hardware technology, several issues still need to be considered regarding heat-dissipation, packaging, and safety before the system can be scaled up for human application. Therefore, our future works will focus on scaling up the system for human application.

## 5. Conclusions and Future Works

This paper presents a new intuitive tele-manipulation scheme for guiding magnetic nanoparticles inside a multi-branch blood vessel by utilizing a haptic device in the human control loop of targeted drug delivery system. Also, a new haptic rendering method for multi-particles nano-manipulation is proposed for steering MNPs to a desired outlet. In user studies, several guidance modes were carried out to evaluate the effectiveness of the proposed scheme. Results showed that, at blood velocities ≤4 mm/s, haptic assistance can significantly improve the targeting performance of a targeted drug delivery system. As a future work, a 3D simulation of the proposed scheme can be studied considering human factors such as operator immersion and satisfaction with the virtual system. 

## Figures and Tables

**Table 1:** Parameters used in simulation studies.

Parameter	Value	Unit
MNP Diameter	400–1000	nm
MNP Density	7200	kg/$m^{3}$
Blood Density	1050	kg/$m^{3}$
Blood Viscosity	0.004	Pa·s
$\mu_{0}$	$4\pi \times 10^{-7}$	$N/A^{2}$
$M_{\mathrm{sat}}$	570.7	kA/m

**Table 2:** Relationship between input current and magnetic field intensity, and magnetic field gradient using a single-coil-core system at the center of the region of interest (ROI).

Current (A)	B (mT)	∇B (T/m)
1	28.2	0.43
2	54.1	0.92
3	79.8	1.39
4	106.3	1.90
5	132.6	2.40
6	160.7	2.8

**Table 3:** Results of two-way analysis of variance.

Analysis Parameter	Factor	F	*p*-Value
Success Rate	Haptic Cues	(2, 22) = 59.529	<0.001
	Blood Velocity	(2.120, 23.321) = 20.240	<0.001
	Interaction	(8, 88) = 2.887	0.007

## Supplementary Materials

The following are available online at www.mdpi.com/2072-666X/9/1/14, Video S1: UG, Video S2: SVF and Video S3: FVF.
